# The Influence of Recycled Materials on Cold Mix with Foamed Bitumen Properties

**DOI:** 10.3390/ma16031208

**Published:** 2023-01-31

**Authors:** Przemysław Buczyński, Juraj Šrámek, Grzegorz Mazurek

**Affiliations:** 1 Department of Transportation Engineering, Faculty of Civil Engineering and Architecture, Kielce University of Technology, Al. Tysiąclecia Państwa Polskiego 7, 25-314 Kielce, Poland; 2 Department of Construction Management, University of Zilina, Univerzitna 8215/1, 010 26 Zilina, Slovakia

**Keywords:** foamed bitumen, cold recycled mixture, recycled material, reclaimed asphalt pavement, reclaimed concrete, recycled aggregate

## Abstract

The utilization of recycled materials is an important issue in the context of environmental protection. The large amounts of recycled material recovered from the demolition of asphalt road structures indicate the need to find new ways of utilizing them. In the case of road renovation projects, large amounts of recycled materials are, in most cases, recovered in the form of reclaimed asphalt pavement (RAP), reclaimed concrete (RC) and recycled aggregate (RA). To focus on the effects of the use of recovered materials (RAP, RC and RA), the same composition was used for all of the analyzed mixtures in terms of foamed bitumen (FB) and Portland cement (CEM) content. The scope of laboratory tests included the specification of the following parameters: the amount of air void content Vm, the determination of axial compression strength at +25 °C, indirect tensile strength (ITS) at +25 °C, water resistance, TSR, water and frost resistance, WR_W+M_ stiffness modulus (IT-CY) at 13 °C, dynamic dynamicmodulus. The plan of the experiment assumed addition recycled material in quantities between 20% and 80% in increments of 20%. The obtained results indicate that both the type and quantity of recycled material significantly affect the properties of the cold-recycled mixture with foamed bitumen. Using reclaimed asphalt pavement and recycled cement concrete guarantees high levels of stiffness in the recycled mixture. Howeverin the case of recycled aggregate, the authors did not observe any visible changes in the dynamicdynamic modulus, irrespective of the loading conditions. It was also indicated that it is necessary to reduce the quantity of reclaimed asphalt pavement in the composition of the FB-RCM mix to maintain the required air void content.

## 1. Introduction

Damaged surfaces require maintenance and reconstruction. Typical maintenance and resurfacing technology usually use hot mix asphalt produced at temperatures above 160 °C (HMA). An alternative to HMA technology is cold recycling technology. In the cold recycling technologies, asphalt emulsions and foamed bitumen bitumen are commonly used as binders. The use of cold recycling technology is beneficial for the environment in terms of: the possibility of using recycled material and lower energy consumption, lower smoke and greenhouse gas emissions [1]. The general idea of the full-depth cold recycling is to maximize the use of materials obtained from damaged layers of the road pavement structure [2,3]. Most road renovation projects that involve the use of full-depth cold recycling technology are carried out on roads with a flexible and semirigid structure [4,5]. It must be noted that local conditions, such as the arrangement of the existing structural layers, determine the proportional content of individual components in the recycled base course mix. The composition of a recycled base course may therefore include: bounded mixtures for base course layers [6] or cement concrete layers [7], non-bound mixtures and asphalt pavements [8,9]. This approach maximizes the use of materials from the existing structural layers in the composition of the base course created in the full-depth cold recycling. The amount of recycled material in the form of reclaimed asphalt pavement (RAP), reclaimed concrete (RC) and recycled aggregate (RA) in the composition of recycled base course depends on the thickness of the structural layers.

The most commonly used recycled material in the composition of recycled cold mixes with foamed bitumen is reclaimed asphalt pavement (RAP). Reclaimed asphalt pavement is usually used in recycled mixtures in the quantities of 20–70%. Reclaimed asphalt pavement in the amount of 20% was used by Niazi and Jalili [10] in their studies, conducted to assess the effect of Portland cement and hydrated lime on the properties of mineral-binder mixes with foamed bitumen. Chomicz-Kowalska [3], in her assessment of compaction methods, used 50% of reclaimed asphalt pavement in the mineral mix. It was shown that the indirect tensile strength (ITS) of unconditioned samples is above 500 kPa regardless of the compaction methods. The effect of the amount of reclaimed asphalt pavement (0%, 50% and 70%) in the composition of a recycled base course with foamed bitumen on the dynamic modulus value was presented by Godenzoni et al. in their paper [11], where limestone aggregate of the granularity of 0/5 mm, 5/10 mm and 10/14 mm was used as a virgin aggregate. Considering all the tested mixtures, the stiffness modulus ranged between 171 MPa and 4075 MPa. This objective was achieved by measuring the dynamicdynamic modulus under uniaxial cyclic compression tests in a range of temperatures (from −20 °C to 55 °C).

The reclaimed cement concrete (RC) is a special case of marginal material used for the recycled cold mixture. Zou et al. [12] presented influence factors on using recycled concrete aggregate in foamed bitumen mixtures based on tensile strength and moisture resistance. The results indicate that 100% reclaimed concrete aggregate as a substitute for natural aggregate can be used in foamed bitumen mixtures for the base and subbase courses of asphalt pavement. It was found that the performance of foamed bitumen mixtures was improved by the cement treatment, but redbrick deteriorated it, and that reclaimed concrete aggregate does not affect the length of the curing time. The stiffness of cold asphalt mixtures (CAM) with 100% recycled construction and demolition recycled aggregates (CDWA) was studied by Gómez-Meijide et al. [13]. It was found that CAM with CDWA frequently achieved higher stiffness than control mixes using natural aggregate (NA), but that they required significantly higher contents of bitumen and water. They were less susceptible to temperature and, therefore, potentially more fatigue-resistant, but more complicated to design. Finally, a clear dependency on the compaction process (static and gyratory) was also found.

Similar to the cement concrete (RC) application, the use of recycled natural aggregate (from the granular base layer) is rare in the composition of the recycled cold mix with foamed bitumen. The study by Iwański [14] presents the results of laboratory testing of the physical and mechanical parameters of the recycled material using the foamed bitumen and its resistance to the action of water. The tests were performed on the road base mixtures incorporating reclaimed asphalt pavement (RAP) with foamed bitumen. The aim of the tests was to evaluate the properties of the mixes in terms of the recycled aggregates. The mixes included aggregates from the recycling of the crushed stone base layer and from the crushed concrete rubble. The analysis of the test results allowed for the statement that if 2.5% of foamed bitumen and 2.0% of Portland cement were used, the recycled road base had the required physical and mechanical properties and moisture resistance.

The objective of this research is to investigate the effect of recycled materials on the properties of cold-mixed foamed bitumen (FB-RCM). The aim of the work is also to determine the impact of varying amounts of recycled components in the range of 20–80% in the composition of the mixture. This case study may contribute to a better understanding of this emerging cold recycling technology with foamed bitumen. The possibility of maximizing the use of recycled materials in the mineral-cement mix with foamed bitumen (FB-RCM) will facilitate the reduction in recycling generated during road renovation projects.

## 2. Purpose and Scope of Research

The aim of this research was to determine the effect of recycled materials from the demolition of damaged road pavement structures, such as reclaimed asphalt pavement (RAP), reclaimed concrete (RC) and recycled aggregate (RA), on the physical, mechanical and rheological properties of recycled base course with foamed bitumen.

To concentrate on the effects of the use of recovered materials (RAP, RC and RA), the same composition was used for all of the analyzed mixtures in terms of foamed bitumen (FB) and Portland cement (CEM) content. Additionally, during the design phase, it was assumed that the mineral composition would be the same in terms of gradation and the design curve. This enables the comparison of the effects of recovered materials on the properties of the mineral-cement mix with foamed bitumen.

**Figure 1:**
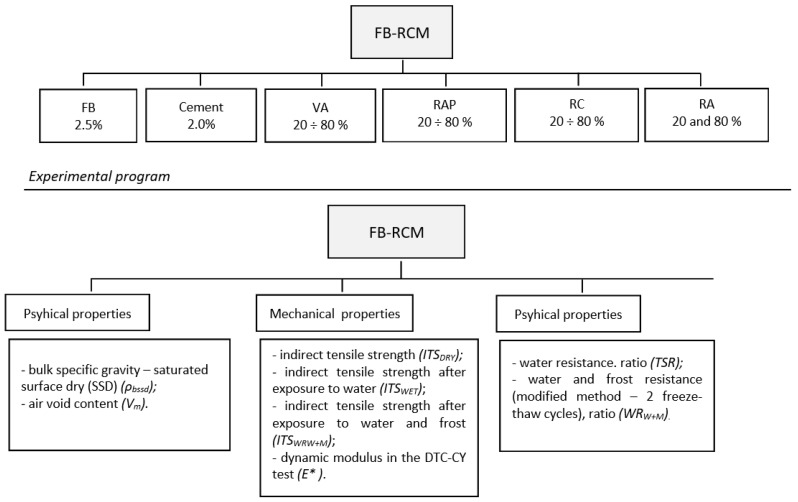
Experimental design.

The scope of laboratory tests included the specification of the following parameters (Figure 1):

- the amount of air void content Vm according to EN 12697-8 [15],
- the determination of axial compression strength at +25 °C according to EN 13286-41 [16],
- indirect tensile strength (ITS) at +25 °C according to EN 12697-23 [17],
- water resistance, TSR according to Wirtgen 2006 [18],
- water and frost resistance WR_W+M_ according to AASHTO T283 [19],
- the determination of the stiffness modulus using the indirect tensile test (IT-CY) at 13 °C according to standard EN 12697-26 Appendix C [20],
- the determination of the dynamicdynamic modulus according to EN 12697-26 Appendix D [20].

## 3. Experiment Plan

### 3.1. Materials

#### 3.1.1. Reclaimed Asphalt Pavement (RAP)

The reclaimed asphalt pavement (RAP) used in the research was created by milling layers of the road surface structure composed of asphalt concrete. The milling included a surface and a binder course layer. The quality of reclaimed asphalt pavement was evaluated during a series of tests that were carried out in accordance with standards PN-EN 13108-8 [21] and WT-2/2014 [22].

The main parameters of the extracted asphalt were determined using a set of recycled material samples. Bitumen from reclaimed asphalt pavement (RAP) was recovered using an extraction method performed in accordance with EN 12697-1 point B.1.2 [23]. A rotary evaporator, according to EN 12697-3 [24], was used to separate the bitumen from the solvent. The results of this analysis are presented in Table 1 and Table 2. The span of the results is shown in the form of a confidence interval, with the assumption of a probability of 95%.

The value of the penetration index (PN-EN 12,591 Appendix A [29]) was determined on the basis of the results of penetration and softening point definition. The value of the penetration index was determined using Equation (1):

(1) $$IP=\frac{20\cdot T_{PiK}+500\cdot \log\left(P\right)-1952}{T_{PiK}+50\cdot \log\left(P\right)-120}$$

where *T_PiK_*—softening point, [°C], log(*P*)—decimal logarithm of the penetration value at 25 °C, [0.1 mm].

The calculated value of the penetration index was determined at *IP* = −0.8

The analysis of the granulometric composition of reclaimed asphalt pavement is presented in Table 2, which is necessary for the evaluation of the uniformity of gradation in accordance with WT-2/2014 [22] and EN 933-1 [30].

On the basis of the conventional tests of asphalt recovered from reclaimed asphalt pavement, the asphalt can be classified in the category declared on the basis of penetration Pdec = 60 [0.1 mm], which conforms with the requirements of category P15 according to WT-2/2014 [22]. The obtained average elastic recovery value prevents this bitumen from being classified as a modified binder. The achieved value of dynamic viscosity suggests that the asphalt is from the lower penetration range of unaged 50/70 asphalt, which would confirm the penetration index result of *IP* = −0.8. The quality assessment of the recovered aggregate primarily indicates a strongly uniform granulometric composition of the reclaimed asphalt pavement.

#### 3.1.2. Recycled Cement Concrete (RC)

Recycled cement concrete is recovered by the crushing process of cement concrete class: C25/30 and C30/37. The quality of recycled cement concrete was assessed on the basis of a comparison of its physical and mechanical properties with the harmonized standard EN 13,242 [31]. Test results attributed to specific categories are presented in Table 3.

#### 3.1.3. Recycled Aggregate (RA)

The tested recycled aggregate (RA) was obtained during the milling of a road structure in which the base course was created with natural crushed aggregate of the gradation 0/31.5 mm. The quality of the aggregate was assessed on the basis of the harmonized standard EN 13,242 [31] in terms of its physical and mechanical properties. Test results related to physical and mechanical properties are presented in Table 4.

#### 3.1.4. Bituminous Binder, Portland Cement

To improve the cohesion of the mineral mix, foamed bitumen (FB) and class CEM I 42.5R Portland cement (PC) were used. Foamed bitumen was generated from 50/70 paving grade bitumen. Foamed bitumen was generated in a Wirtgen WLB10S bitumen foaming device. The properties of paving grade bitumen are presented in Table 5, while the properties of CEM I 42.5R Portland cement are shown in Table 6.

**Figure 2:**
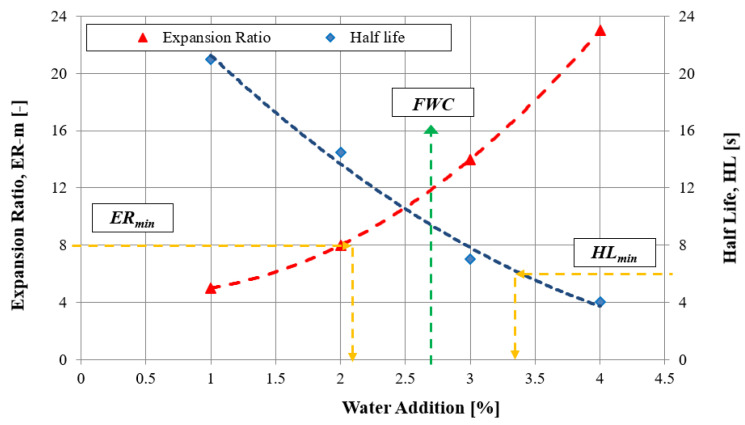
Characteristics of foamed bitumen for 50/70 paving grade bitumen.

The suitability of 50/70 paving grade bitumen was established in accordance with the experiment plan and in accordance with the methodology implemented by the authors of the papers [41,42,43]. Bitumen 50/70 is a typical bitumen used to produce foamed bitumen [44]. The optimum amount of water needed to obtain the foamed bitumen was determined in accordance with the requirements specified in [45]. The results of the assessment are presented in Figure 2.

#### 3.1.5. Virgin Aggregate 0/31.5 (VA)

The virgin aggregate VA, with a grain size of approximately 0/31.5 mm, was also used. The material used was excavated and processed in a limestone mine located in the Świętokrzyskie. The virgin aggregate VA requirements were specified in the guidelines [46]. The properties of virgin aggregate (VA) were the same as those of recycled aggregate (RA) (Table 4). The RA and VA aggregates were limestone.

#### 3.1.6. FB-RCM Mix Design

The design of the recycled base course composition included the achievement of a mineral-cement mix gradation curve that would ensure the fulfillment of the optimum gradation requirement. The optimum shape of the gradation curve of the recycled mix corresponded with the recommendations for full-depth cold recycling [45].

The design of the mineral mix with foamed bitumen (FB) intended for the cold recycled mix (RCM) was prepared using different amounts of recycled materials. The amount of recycled materials (RAP, RC and RA) included in the mix was selected from the range between 20% and 80% in increments of 20%. This assumption was implemented during the composition design for reclaimed asphalt pavement (RAP) and recycled cement concrete (RC). A different approach was implemented in the case of recycled aggregate (RA). The same proportioning range was implemented, namely between 20% and 80%, but only limit values were included in the experiment plan. It resulted from the similar physical and mechanical properties of the granulating aggregate (VA) to the recycled aggregate (RA). Similarities in properties indicated the same effect of these two components on the properties of the FB-RCM blend. Therefore, the content of these components was limited. The proportional content of all components of the mineral-cement mixes with foamed bitumen, with respect to the amount and type of materials recovered from the pavement, is shown in Table 7, Table 8 and Table 9.

**Figure 3:**
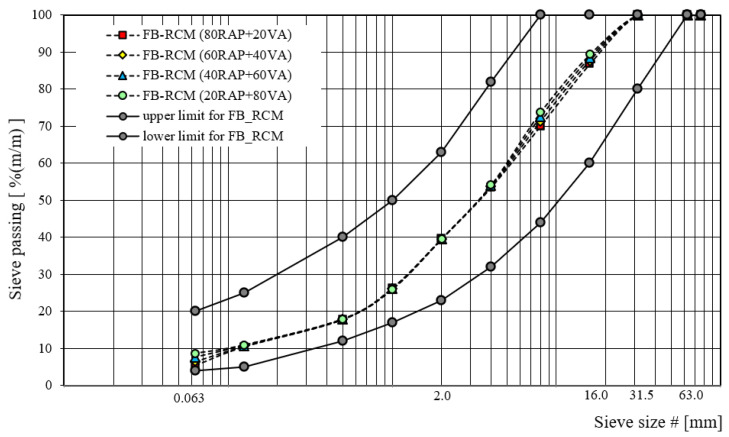
Gradation curve for FB-RCM with RAP.

**Figure 4:**
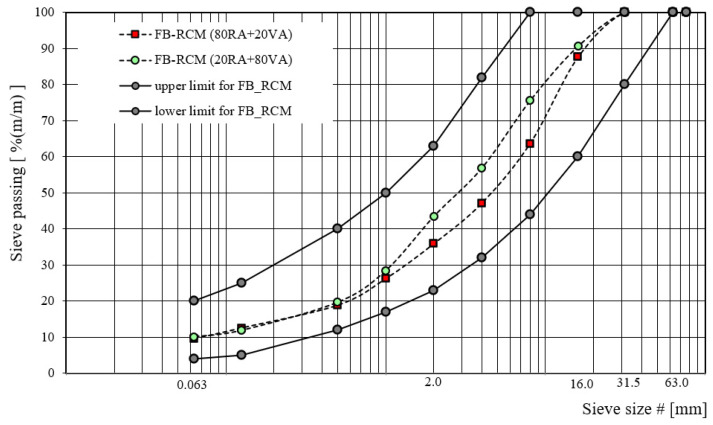
Gradation curve for FB-RCM with RA.

**Figure 5:**
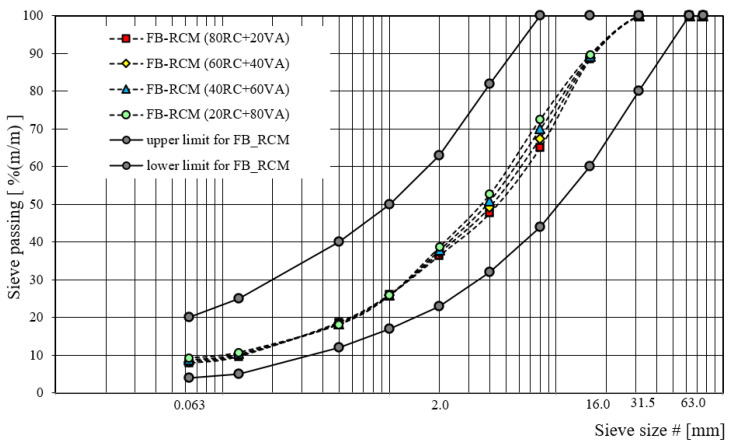
Gradation curve for FB-RCM with RC.

The graphic visualization of designed gradation curves for recycled base-course aggregate mixtures with foamed bitumen, in terms of recycled aggregate type, is shown in Figure 3, Figure 4 and Figure 5.

The next phase of the FB-RCM mix design process consisted of determining the optimum moisture content (OMC). The optimum amount of water in the mineral mix was determined in accordance with standard PN-EN 13286-2 [47], using the proctored method. The results of the research are presented in Table 10 for each type of recycled material.

#### 3.1.7. Preparation of the Samples and Compaction

The tested samples were prepared in laboratory conditions in a WLM30 mixer, while foamed bitumen was generated in a WLB10S device. To minimize the effect of any variations in quantities of components due to their moisture content, they were initially dried to a fixed mass. In the case of RAP, the drying process took place in a dryer at a temperature of 40 °C, which is below the softening point of the bitumen present in reclaimed asphalt pavement. In the case of aggregate, the temperature was 105 °C.

The components of the mineral mix were mixed in a suitable WLM30 laboratory mixer, which is compatible with the WLB10S laboratory foamed bitumen generator and enables the introduction of bitumen foamed bitumen during the mixing of the components. The benefit of this process is that it enables the simulation of the full-depth cold recycling process with foamed bitumen in real-world conditions.

Upon the generation of a uniform mix, the samples were compacted. Samples designated for the testing of the dynamicdynamic modulus were compacted in a gyratory compactor in accordance with the requirements of standard 12697-31 [48]. The settings of the gyratory press were determined on the basis of previous research [11,49,50]. The number of gyrations for compaction was chosen individually for the mixes, to achieve densities at which the air void content in the mineral-cement mix with foamed bitumen equals Vm = 12.0%. The diameter of the samples of recycled mix with foamed bitumen (FB-RCM) prepared for testing was D = 150 mm, and their height was H = 180 mm.

Samples designated for the testing of the main physical and mechanical properties were compacted in a static press with a load of 80 kN and a compaction time of 3 min [46].

## 4. Testing Methods

Testing methods used to determine the properties of the FB-RCM mix were selected on the basis of an analysis of Polish and international research [18,45,51,52,53,54]. The plan for the testing of the cold recycled mix with foamed bitumen (FB-RCM) included the determination of the main physical and mechanical properties in accordance with the scope presented in Table 11. The number of measurements depended on the requirements of the test methods. The minimum number of samples needed to determine the mean value was five.

### 4.1. Air Void Content

Air void content *V_m_* [15] is the volume of air pores in the sample of the mineral mix with foamed bitumen, expressed as a percentage of the total sample volume, in accordance with the following formula:

(2) $$V_{m}=\frac{\rho_{m}^{}-\rho_{b}}{\rho_{m}}\cdot 100\%$$

where *ρ_m_* is the density of the mineral mix with foamed bitumen and mineral dust [Mg/m^3^], and *ρ_b_* is the bulk density of the mineral mix with foamed bitumen and mineral dust [Mg/m^3^].

The amount of air void content is determined using the density *ρ_m_* and bulk density *ρ_b_* specified below.

The bulk density *ρ_b_* of the mineral mix with foamed bitumen and recycled materials (RAP, RC, RA) was determined using the traditional hydrostatic method in accordance with EN 12697-6 [55] according to the following formula:

(3) $$\rho_{b}^{}=\frac{m}{m_{2}-m_{1}}\cdot \rho_{w}$$

where *m* is the mass of the dry sample [g], *m*_1_ is the mass of the sample in water [g], *m*_2_ is the mass of the sample in the air after being removed from water [g], and *ρ_w_* is the density of water at test temperature [Mg/m^3^].

The density *ρm* of the mineral mix with foamed bitumen and mineral dust was determined using the mathematical method described in EN 12697-5 [56].

(4) $$\rho_{m}=\frac{100}{\left(p_{a1}/\rho_{a1}\right)+\left(p_{a2}/\rho_{a2}\right)+\left(p_{a3}/\rho_{a3}\right)+\left(p_{b}/\rho_{b}\right)}$$

where *p_a_*_1_—proportional quantity of virgin aggregate (VA) in the mineral mix with foamed bitumen [%], *ρ_a_*_1_—density of virgin aggregate grains [Mg/m^3^], *p_a_*_2_—proportional quantity of (RAP, RC, RA) in the mineral mix with foamed bitumen [%], *ρ_a_*_2_—density of (RAP, RC, RA) grains [Mg/m^3^], *p_a_*_3_—proportional quantity of Portland cement in the mineral mix with foamed bitumen [%], *ρ_a_*_3_—density of Portland cement grains [Mg/m^3^], *p_b_*—proportional content of foamed bitumen in the mixture [%], *ρ_b_*—density of foamed bitumen [Mg/m^3^], *p_a_*_1_ *+ p_a_*_2_ *+ p_a_*_3_ *+ pb* = 100% (m/m).

### 4.2. Axial Compression Strength

Axial compression strength (RC) was tested on cylindrical samples that were compacted according to the Marshall method [57]. Compression tests were carried out at a temperature of 25 ± 3 °C, in compliance with the requirements of standard EN 13286-41 [16]. The compression strength was determined for samples after 28 days of curing. The compression strength was calculated in accordance with Equation (4).

(5) $$R_{C}=\frac{F}{A_{C}}$$

where *R_C_*—compression strength of samples made of mixtures bound with cement (N/mm^2^), *F*—maximum transferred force (N), and *A_C_*—cross section area of a sample composed of mixture bound with cement (mm^2^).

### 4.3. Indirect Tensile Strength

Indirect tensile strength ITS_DRY_ [17] of the cold recycled mix with foamed bitumen was tested on Marshall samples with a diameter of 101.6 ± 0.3 mm and a height of 62.5 ± 2.5 mm, cured for 28 days with optimum moisture content (OMC). The tests were carried out at a temperature of 25 °C. Tests were performed by placing the samples between two plates and compressing them with a constant strain rate of 50 ± 2 mm/min. Indirect tensile strength ITS was calculated in accordance with Equation (6).

(6) $$ITS_{DRY}=\frac{2\cdot P}{\pi \cdot h\cdot D}$$

where *P*—maximum sample breaking force, *h*—height of sample and *D*—diameter of sample [mm].

### 4.4. Weather Resistance

#### 4.4.1. Water Resistance (TSR)

The assessment of water resistance is the most important parameter that determines the quality of a road’s base course. Due to its location in a road structure, the base course layer can be subject to constant dampness and exposure to ground and storm water. The absence of direct sunlight extends the drying time of the base course.

The resistance of the base course was determined using the methodology described in [18], which resulted in the achievement of a TSR index value. The TSR index—tensile strength retained—was established by regulations [18,45] and represents the loss of strength during indirect tensile tests exposed to water in comparison with tests not exposed to water and is expressed by the following formula:

(7) $$TSR=\frac{ITSwater}{ITS}$$

where *ITS_water_*—indirect tensile strength at the temperature of 25 °C of samples subject to conditioning [kPa], *ITS*—indirect tensile strength at the temperature of 25 °C of samples not subject to conditioning [kPa],

To determine the loss of strength connected with the negative effect of water, samples must be left in the water for 24 h at a temperature of 25 ± 1 °C. After their removal from the water, they must be superficially dried and tested for indirect tensile strength at a temperature of 25 °C. A base course is considered water resistant, i.e., watertight, if the value of its TSR index is ≥0.7 in accordance with [18,58].

#### 4.4.2. Water and Frost Resistance (WR_W+M_)

Resistance to water and frost were determined using the procedure described in American Standard AASHTO T283 [19]. The testing procedure consists of the assessment of 18 freezing cycles in order to determine the most unfavorable operating conditions of the structure. Each freezing and thawing cycle simulates the autumn/winter and spring/winter operating periods, characterized by high temperatures and thawing during the day and below-zero temperatures and freezing at night.

Tests were carried out on Marshall samples of the following dimensions: Ø = 101.6 ± 0.1 mm, height h = 63.5 ± 5 mm. The prepared test samples achieved a compaction index of 98%. Tests were carried out after 28 days of curing. They were performed in a Marshall press with a piston speed of 50 mm/min and using load rings with a width of 12 mm and a curvature of 50.5 mm that transferred the force onto the sample until its destruction.

After the forming of the samples, they were divided into two groups and underwent different curing:

- The control group (without curing) was stored in the laboratory at ambient temperature, until the moment of the first indirect tensile strength test.
- Curing simulated exposure to water and frost. The second group underwent the B curing process (resistance to water and frost), which was similar to the modified procedure [59]. The samples were stored in water at a temperature of 20 °C and under a negative pressure of 200 hPa for 30 minutes. They were then placed in a water bath at a temperature of 60 °C and were kept there for 24 h. The next phase consisted of two freezing-defrosting cycles (−18 °C for 4 h, 20 °C for 4 h).

Immediately before the test, it was necessary to bring all samples to the same testing temperature, defined as [60] –25 °C. The criteria for water resistance WR_W+M_ and for water and frost resistance WR_W+M_ are determined according to the following formula:

(8) $$WR_{W+M}=\frac{ITS_{W+M}}{ITS}\cdot 100\%$$

where *WR_W_*_+*M*_—indirect tensile strength index for the curing method of exposure to water and frost [%], *ITS_W+M_*—indirect tensile strength of cured samples exposed to water and frost [kPa] and *ITS*—indirect tensile strength of samples stored in dry air conditions [kPa].

The observed increase in the WR_W+M_ value indicates the resistance of the recycled base course to water as well as to water and frost. On the basis of research [61,62,63] by Iwański, the values of the WR_W+M_ index were considered acceptable when they exceeded 70%.

### 4.5. Stiffness Modulus (Sm)

The value of the modulus of rigidity was determined based on the indirect tension tests (IT-CY), carried out in accordance with the requirements of standard EN 12697-26, Appendix C. This test includes the measurement of vertical and horizontal displacement in the middle of the sample’s height and the monitoring of the applied force that generates the intended displacement. The horizontal displacement of the sample was 5 ± 2 μm, with the diameter of the tested samples equaling 101.6 mm. The duration of the force increase should last for 124 ± 4 ms. The modulus of elastic rigidity can be calculated with Equation (8), while the value of Poisson’s ratio can be calculated from Equation (9):

(9) $$Sm=\frac{F\cdot \left(\nu +0.27\right)}{z\cdot h}$$

(10) $$\nu =3.59\cdot \frac{z}{\Delta V}-0.27$$

where *S_m_*—the modulus of rigidity of sample, [MPa], *F*—the maximum force applied to sample, [N], *v*—temperature-dependent Poisson’s ratio, *z*—the amplitude of horizontal displacement of sample during loading, [mm], *h*—thickness of sample, [mm] and Δ*V*—the maximum vertical displacement of sample (corresponding to its highest horizontal displacement) [mm].

Upon the analysis of Equation (9) we can conclude that higher transverse deformation and thickness of the tested sample result in lower internal stress in the pavement and therefore lower values of the modulus of rigidity Sm. In cases where the loading surface ratio is different than 0.6, the modulus of rigidity is calculated using Equation (10):

(11) $$S_{m}=S_{m}{}^{\prime }\cdot \left[1-0.322\cdot \left(\log(S_{m}\right)-1.82\right)\cdot \left(0.60-k\right)]$$

where *Sm*—the modulus of rigidity [MPa] and *k*—the measured value of the loading surface ratio.

The value of the modulus of rigidity was tested at a temperature of 13 °C, following a 28-day conditioning period.

### 4.6. DynamicDynamic Modulus |E*|

The dynamicdynamic modulus (absolute value of compex modulus E*) of the cold recycled mineral bitumen mix with foamed bitumen was determined on the basis of a DTC-CY (direct tension-compression test on cylindrical samples) test, in accordance with the requirements of standard EN 12697-26 App. D [20]. In accordance with the direct tension-compression method (DTC-CY), the sample is subjected to cyclical sinusoidal loads that generate minor deformation in the range of 25–50 με [11,20,64]. Tests performed beyond this deformation range may cause the sample to deform irreversibly, which may induce errors in the interpretation of the dynamic modulus. The tests were performed with four temperature values (5 °C, 13° C, 25 °C and 40 °C) and six frequencies (0.1 Hz, 0.3 Hz, 1 Hz, 3 Hz, 10 Hz and 20 Hz). On this basis, the authors determined the value of the dynamic modulus (E*) and the phase shift angle (φ).

Following an analysis of the influence of materials from the smelting and steel industries on the properties of a cold recycled mineral-bitumen mix with foamed bitumen, the authors proposed Richard’s asymmetric model [65], which represents a modification of the model in the NCHRP report 9-29: PP 02 [66,67]. This model can be classified as a sigmoidal, asymmetric mathematical model with the introduction of the curve asymmetry coefficient (λ). The sigmoidal asymmetric function has been defined by Equation (12):

(12) logE*=δ+α1+λeβ+γlogω1λ

where E*—dynamic modulus [MPa], *ω*—temperature shift factor, *δ*—value of the bottom asymptote (master curve adaptation parameter), α—difference between the value of the top and bottom asymptote (master curve adaptation parameter), *λ*, *β*, *γ*—master curve adaptation parameters.

The master curve of the dynamic modulus was presented using the principle of time-temperature superposition. This requires the introduction of a temperature shift factor (αT) in the form of a polynomial function (13) [68]:

(13) $$ln\alpha_{T}=a+b\cdot T+c\cdot T^{2}$$

where $\alpha_{T}—$shift factor, T—test temperature [°C] and a,b,c—model parameters.

In the aspect of material type, the principles of time-temperature superposition were implemented during the process of construction of master curves for the dynamic modulus of recycled mixtures with foamed bitumen [67]. For this purpose, the sigmoidal function was optimized using the method of minimizing the sum of squares of deviations for the defined dynamic modulus of the recycled mix with foamed bitumen. As a result of the optimization of the function, it was necessary to define the parameters of the master curve (*α*, *β*, *γ*, *δ* and *λ*). The quality of the adaptation of the sigmoidal function was evaluated using the criterion of the root mean square deviation error (RMSE), which was implemented by the authors of [69].

## 5. Test Results

### 5.1. Test Results of the Research on the Effect of Recycled Materials on Physical and Mechanical Properties

**Figure 6:**
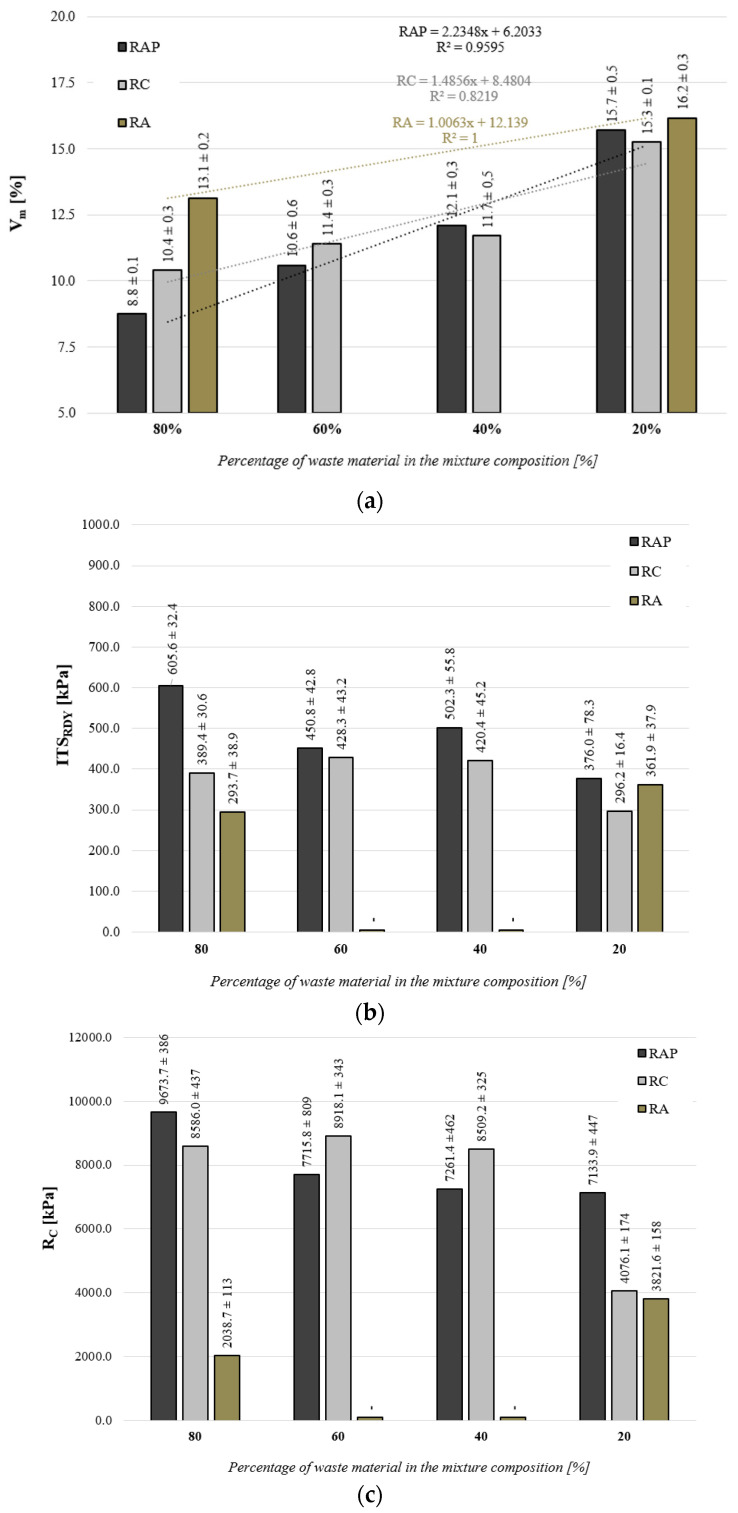
Comparison of test results for: (**a**) air void content (Vm), (**b**) indirect tensile strength (ITSDRY), (**c**) axial compression strength (RC), confidence interval of 95%.

In accordance with the implemented test plan, the following properties were analyzed in the context of the assessment of the effect of the quantity and type of recycled materials (RAP, RC, RA): air void content (Vm), indirect tensile strength (ITS) and axial compression strength (RC). The graphic interpretation of the test results is shown in Figure 6. The spread of the results is shown in the form of a confidence interval, with the assumption of a probability of 95%.

An analysis of the impact of the quantity and type of recycled material (RAP, RC and RA) indicates that in the context of the analyzed values, both the quantity and type of recycled materials affect the main physical and mechanical properties. An analysis of the air void content (Vm) indicates that the reduction in the quantity of virgin aggregate in the composition of the FB-RCM allows for the achievement of a tighter internal structure of the FB-RCM mix. This correlation was also observed irrespective of the type of recycled material used. Increasing the amount of recycled material (RAP, RA and RC) in the composition of the FB-RCM mix has a variable effect on the value of Vm. The fastest rate of increase in air void content was observed in the case of reclaimed asphalt pavement (RAP). Reducing the amount of RAP from 80% to 20% almost doubles the amount of air void content in the mix. In the case of mixtures containing 20% of reclaimed asphalt pavement, the amount of air-void content equals 15.7%, while in the case of a mixture containing 80% of recycled material, the achieved amount of air void content equals 8.8%. It must be noted here that the optimum amount of air void content, in the middle of the range specified in regulation [51], i.e., between 8% and 15%, was achieved with the content of reclaimed asphalt pavement between 40% and 60%. Too much reclaimed asphalt pavement in the FB-RCM mix may cause that FB-RCM mixture become too compacted and will be characterized by a reduced air void, which may be very susceptible to deformation. Similar correlations were observed for the other analyzed recycled materials composed of cement concrete and aggregate, but the effect of increasing their amount in the FB-RCM mix was not as significant as in the case of RAP. The lowest increase was observed for recycled aggregate. The amount of air-void content changed by only 3.1%. This is because RA material is a natural aggregate whose properties are similar to those of the virgin aggregate, and the differences may have been caused by the gradation of the mix and the obtained mineral structure. FB-RCM mixtures containing recycled aggregate are mixtures that have been deprived of the main ingredient in the form of reclaimed asphalt pavement [18,52,53]. The absence of reclaimed asphalt pavement (RAP) in the FB-RCM mix will result in higher air void content, which was confirmed by the results of tests for air void content in the FB-RCM mix with 20% and 80% recycled aggregate. Comparing the obtained results of air voids (Vm), it should be noted that the minimum air voids were obtained for mixtures with a maximum content of marginal materials equal to 80%. For FB-RCM with RAP, it was 8.8%, and for reclaimed cement concrete (RC) with recycled aggregate (RA), it was 13.1%. In contrast, the highest air void was found for the FB-RCM with 20% recycled material (RAP, RC and RA), the average values of which were approximately 15%.

The analysis of the results of indirect tensile strength (ITS_DRY_) tests reveals that all FB-RCM mixtures, irrespective of the type and quantity of recycled material, demonstrate higher strength than the minimum strength for BSM mixtures (ITS ≥ 250 kPa, a value attributed to a composition containing 50% RAP and 50% of virgin aggregate [18]). One can observe that the results are affected by the type of recycled material. Additionally, in the case of an FB-RCM mix with reclaimed asphalt pavement, it was observed that the amount of recycled material in the mix composition affects the value of its indirect tensile strength. The highest results in indirect tensile strength by far were achieved by mixtures with reclaimed asphalt pavement across the entire recycled material dosage range. In the range of the proportional content of reclaimed asphalt pavement between 40% and 60%, which is optimum in terms of the air void content, the mixtures demonstrate indirect tensile strength between 450 and 500 kPa. The obtained value of breaking stress is higher than the value of the tensile stress that is present in pavement structure specified in the catalog [70]. Indirect tensile strength is a good parameter that enables the protection of the structure against the loss of fatigue strength [2]. The maximum value of indirect tensile strength, equal to 605 kPa, was achieved for the FB-RCM mixture containing reclaimed asphalt (RAP) in the amount of 80%. Comparing the obtained results of the indirect tensile strength of FB-RCM mixtures, in terms of the type of recycled material, it should be stated that reclaimed asphalt provides the opportunity to obtain the highest ITS_DRY_ values. Compared to mixes containing reclaimed cement concrete, the strength is higher in the range from 20 kPa to 220 kPa. In the case of mixes with reclaimed aggregate (RA), ITS_DRY_ is higher and ranges from 15 to 312 kPa. The greatest differences were observed for the highest share rate of recycled materials usage. Upon the assessment of the effect of recycled cement concrete (RC) on the value of ITS, one can observe the stabilization of the parameter at a constant level in the middle of the dosage range. The lowest values of ITS were recorded for the minimum amount of RC material in the FB-RCM mix. In the case of the addition of recycled aggregate (RA), no significant changes in the indirect tensile strength were observed. The values obtained with this type of recycled material were by far the lowest in the analyzed group. A similar impact of recycled materials was observed during the analysis of axial compression strength (R_C_). The effects of the amount and type of material are very similar in terms of correlation, except for the fact that 40% RC and 60% RC mixtures demonstrate the highest values of compression strength. The maximum compressive strengths were obtained for the FB-RCM with the maximum amount of reclaimed cement concrete (RC), and this relationship correlates with changes in indirect tensile strength (ITS_DRY_). FB-RCM mixtures containing RAP and RC have an average compressive strength of 7.7 MPa. The compressive strength of FB-RCM mixtures with recycled aggregate (RA) is almost three times lower than in the case of mixtures with RAP and RC.

### 5.2. The Results of Testing the Effect of Recycled Materials on Water and Frost Resistance

The assessment of resistance to weather conditions is very important in the context of the evaluation of a new material. Exposure to water and frost is one of the factors that can destroy a road structure [71].

Resistance to weather conditions was evaluated based on the assessment of the loss of indirect tensile strength of FB-RCM mix samples that were subjected to the process of conditioning. The effect of recycled materials on the resistance to weather conditions was determined by defining the following properties:

- water resistance TSR [18],
- water and frost resistance WR_W+M_ according to AASHTO T 283 [19].

**Figure 7:**
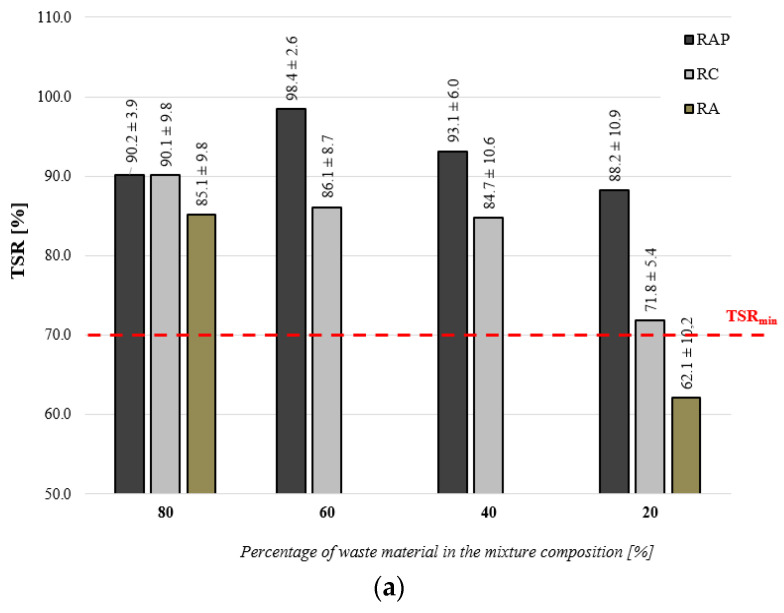

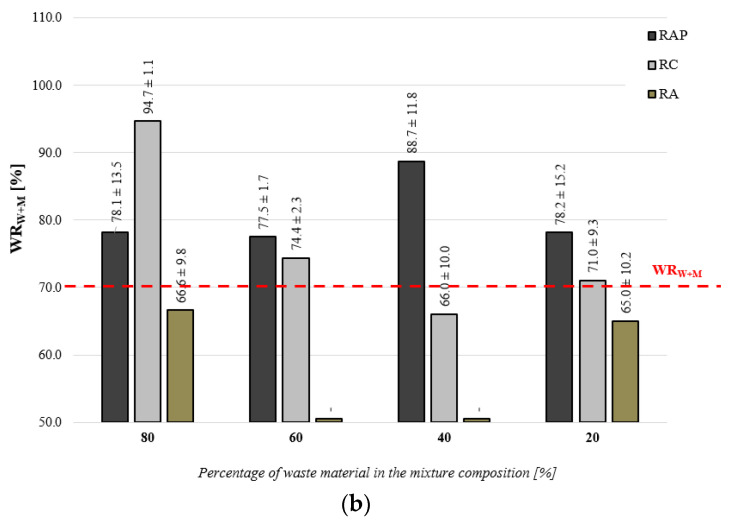
Resistance to water and to water and frost: (**a**) TSR index, (**b**) WR_W+M_ index.

The results of tests performed on FB-RCM mixtures with recycled materials in the form of RAP, RC and RA are shown in Figure 7. The results are shown as mean values with a confidence interval of 95%.

Following an analysis of the test results shown in Figure 7, one can observe that irrespective of the type and quantity of recycled material in the FB-RCM mix, there is no visible loss of indirect tensile strength of samples conditioned in water. All mixtures, except for the mix with recycled aggregate in the quantity of 20%, demonstrate the minimum resistance to water above the critical value of TSR > 70% [18]. The recycled FB-RCM mixture with RAP is characterized by the highest resistance to water (TSR). The highest water resistance equaled 98% and was observed where RAP was added in the amount of 60%. Lower water resistance than that of RAP was attained with FB-RCM mixtures with reclaimed cement concrete (RC) up to 10%. The lowest TSR = 62% was obtained for the FB-RCM mixture with recycled aggregate in the amount of 20%.

Resistance to water and frost in the form of the (WR_W+M_) index indicates a more variable influence of recycled materials on the durability of FB-RCM mixes. The introduction of an additional destructive factor in the form of exposure to below-zero temperatures resulted in a visible loss of resistance to water and frost and therefore allowed the negative impact of recycled materials on the durability of FB-RCM mixes to be demonstrated. The highest resistance to water and frost was observed for mixtures containing reclaimed cement concrete (RC) in the amount of 80%, where the result of WR_W+M_ was equal to 94%. However, the highest durability, in terms of exposure to water and frost, was demonstrated by FB-RCM mixtures that contained reclaimed asphalt pavement (RAP). This demonstrates that the use of RAP in FB-RCM mixes represents the correct direction in terms of the utilization of this recycled material. The highest loss of indirect tensile strength, following the conditioning process, was registered in the case of FB-RCM with recycled aggregate (RA) at WR_W+M_ = 66%. Therefore, the use of this recycled material requires a higher level of caution.

### 5.3. Definition of the Stiffness Modulus Using the Indirect Tension Test

**Figure 8:**
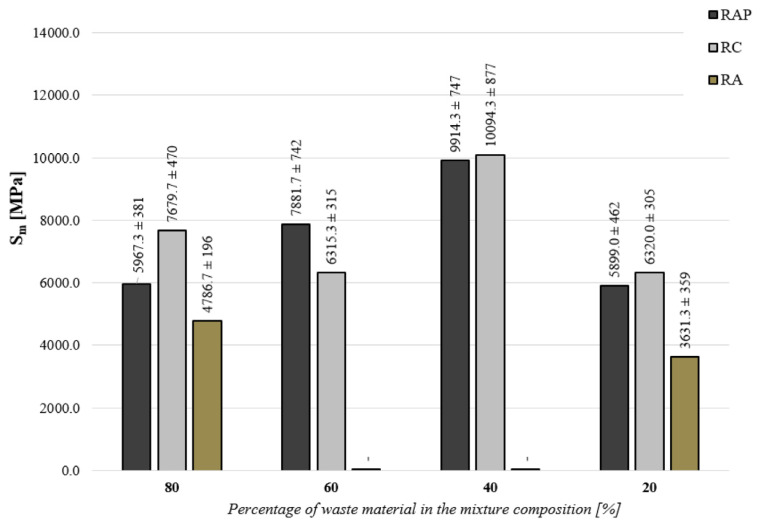
Stiffness modulus determined by indirect tension S_m_ for cold recycled mix in terms of the quantity and type of recycled material.

The graphic interpretation of the test results is shown in Figure 8. The spread of the results is shown in the form of a confidence interval, with the assumption of a probability of 95%. A confidence interval was calculated for the tested number of samples (i.e., *n* = 6). The coefficient of variation with a value below 10% was obtained for all the analyzed FB-RCMs.

The modulus of rigidity of the cold recycled mix with foamed bitumen (FB-RCM) varies depending on the type of recycled material and its proportional amount in the mix. The observed variations in the modulus of rigidity correspond with the origin of the recycled material. Recycled materials that contain bitumen binder and hydraulic binder affect the value of the modulus of rigidity more than recycled aggregate (RA). The maximum value of the stiffness modulus was observed for the FB-RCM mixture with 40% RC content, equal to 10,094 MPa. The FB-RCM blend with 40% RAP content also achieved a high modulus value of 9914 MPa. In all FB-RCM mixtures, at the minimum percentage of recycled materials, i.e., 20%, the value of the stiffness modulus (S_m_) was lowered by half.

The biggest differences in the modulus of rigidity at a temperature of 13 °C were observed when using reclaimed asphalt pavement (RAP). Increasing the amount of reclaimed asphalt pavement in the FB-RCM mix increases the total amount of bitumen binder, which effectively lowers the value of the modulus of rigidity. The observed correlation in the form of the simultaneous reduction in the modulus of rigidity and the increase in the amount of RAP in the FB-RCM mix is confirmed by the test results presented in the paper [72]. The highest rigidity was demonstrated by the recycled mix that contained recycled cement concrete (RC). As demonstrated by the authors of [73], this observation can be explained by the fact that small particles easily react to moisture present in the air and to moisture needed for mix compaction, which generates the effect of secondary hydration. The lowest value of the modulus of rigidity was demonstrated by FB-RCM mixes that contained recycled aggregate. This can be explained by the fact that this recycled material does not contain an additional factor in the form of bitumen or hydraulic binder that guarantees higher cohesion in the mix and therefore provides it with higher rigidity. The results obtained for mixtures that contain recycled aggregate (RA) are representative of a situation in which the FB-RCM mix contains only mineral aggregate.

### 5.4. The Assessment of the Effect of Recycled Materials on Rheological Properties

**Figure 9:**
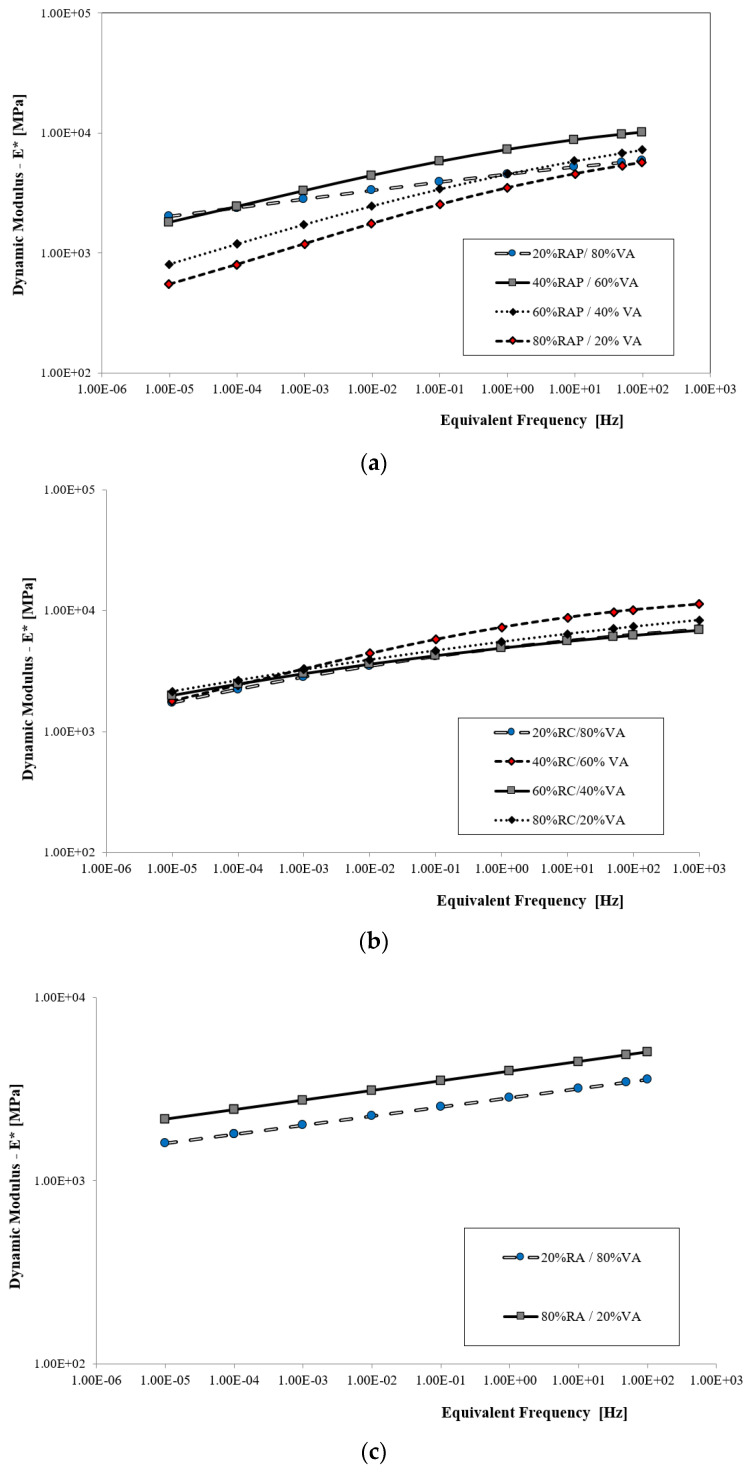
Master curves of the dynamic modulus E* at a temperature of 13 °C for FB-RCM mixtures with (**a**) reclaimed asphalt pavement (RAP), (**b**) recycled cement concrete (RC) and (**c**) recycled aggregate (RA).

The properties of viscoelastic FB-RCM mixtures were assessed based on the dynamic modulus and phase shift angle values. Test results for the dynamic modulus (E*) and the phase shift angle (φ) were obtained in the course of a direct tension-compression test of cylindrical samples (DTC-CY) performed in accordance with the procedure described in standard PN-EN 12697-26 [20], and the resulting values were presented in the form of master curves of the dynamic modulus. The results of these tests are shown in Figure 9.

An analysis of the test results (Figure 9) reveals significant differences in the influence of recycled material (RAP, RC and RA) on the viscoelastic properties of FB-RCM mixtures. The biggest differences were observed in the case of the application of reclaimed asphalt pavement (RAP) in the mix composition. Increasing its amount in the composition intensifies the changes in the elastic properties in the conditions of long load time (less than 1 Hz). The lowest rigidity across the whole reduced frequency spectrum was demonstrated by the FB-RCM mix designated as 80% RAP, i.e., a mix that contained 80% of reclaimed asphalt pavement. This can be explained by the fact that the amount of RAP in the FB-RCM mix increases the total amount of binder and increases flexibility. The mix with the lowest amount of reclaimed asphalt pavement, 20% RAP, was the least susceptible to the loading time changes and to changes in the temperature. The loss of rigidity at low and high frequencies (between 0.01 Hz and 10 Hz) was approx. 1900 MPa. In the case of the mix containing the biggest amount of reclaimed asphalt pavement, the difference is greater and amounts to 3500 MPa. A reduction in the quantity of RAP material generates higher values of the dynamic modulus at low and high frequencies. The increase in the value of the dynamic modulus is not proportional. A higher dynamic modulus increase rate was observed at lower frequencies. In conclusion, it must be noted that, considering the test results obtained for the physical and mechanical properties, the optimum content of reclaimed asphalt pavement in the FB-RCM mix is between 40 and 60%. Mixtures with this amount of recycled material demonstrate the most favorable properties across the whole temperature range.

The assessment of the effect of recycled cement concrete (RC) and recycled aggregate (RA) on the viscoelastic properties of the FB-RCM mix has revealed that their impact is different than that of reclaimed asphalt pavement (RAP). The change in the value of the dynamic modulus in the case of the mix with recycled aggregate is linear and decreases with the progress of the loading time. This tendency is a result of the FB-RCM mix composition and the absence of an asphalt binder that is susceptible to variable load. Asphalt is responsible for increasing the flexibility of the FB-RCM mix, which could be observed in mixes containing reclaimed asphalt pavement RAP. Master curves were defined using the sigmoidal function described by Equation (11) and using the temperature shift factor defined by Equation (12). The values of model adaptation parameters are shown in Table 12.

It must be noted that the adaptation of the mathematical model parameters of an asymmetric sigmoidal function generates a very high R^2^ coefficient, with a value close to 1.0 (Table 12). The achieved RMSE error value is below 5%, which according to the NCHRP 614 report [66] suggests a very good adaptation of the function to the analyzed dynamic modulus values, shifted by the value of the αT factor. An analysis of the master curve “λ” parameter reveals the symmetrical distribution of the dynamic modulus values at low and high frequencies for the analyzed mineral-bitumen mixes with foamed bitumen. It has been observed that asymmetry is reported for mixtures that contain reclaimed asphalt pavement.

## 6. Conclusions

The tests and analyses carried out in relation to the use of recycled materials in the composition of a cold recycled mix, have allowed the authors to formulate the following conclusions:

- Increasing the proportional amount of recycled material in the composition of a cold recycled mix with foamed bitumen reduces the amount of air void content in the mix, irrespective of the type of recycled material used for the obtained grain size of the designed FB-RCM mixes. Comparing the obtained results of air voids (V_m_), it should be noted that the minimum air voids were obtained for mixtures with a maximum content of marginal materials, equal to 80%. For FB-RCM with RAP, it was 8.8% and for reclaimed cement concrete (RC) with recycled aggregate (RA), it was 13.1%. In contrast, the highest air void was found for the FB-RCM with 20% recycled material (RAP, RC and RA), the average values of which were approximately 15%.
- The content of reclaimed asphalt pavement in the FB-RCM mix should be limited to between 40% and 60%. The reason for this is the need to ensure an optimum amount of air void content, which in the case of its proportional content below or above the proposed range exceeds the acceptable values of the air void content parameter, i.e., between 8% and 15%.
- The FB-RCM mixture contained 40–60% of reclaimed asphalt pavement (RAP) and demonstrated indirect tension strength in the range between 450 kPa and 500 kPa. The maximum value of indirect tensile strength, equal to 605 kPa, was achieved for the FB-RCM mixture containing reclaimed asphalt (RAP) in the amount of 80%.
- The maximum compressive strengths (R_C_) were obtained for the FB-RCM with the maximum of reclaimed cement concrete (RC) and this relationship correlates with changes in indirect tensile strength (ITS_DRY_). FB-RCM mixtures containing RAP and RC have an average compressive strength of 7.7 MPa.
- FB-RCM mixtures with recycled aggregate (RA) demonstrate the lowest resistance to water and frost WR_W+M_ = 65%.
- The highest resistance to water (TSR = 98%) and the water and frost resistance (WR_W+M_ = 88%) were observed where RAP was added in the amount of 40 and 60%.
- The maximum value of the stiffness modulus was observed for the FB-RCM mixture with 40% RC content equal to 10,094 MPa and 40% RAP content equal to 99,143 MPa.
- Increasing the amount of reclaimed asphalt pavement in the FB-RCM mix intensifies the changes in the elastic properties in conditions of long load time (below 1 Hz).

## Figures and Tables

**Table 1:** Results of conventional tests of asphalt extracted from recycled material with confidence intervals.

Asphalt Parameter	Unit	Result	Confidence Interval	Range
Qty of binder, EN 12697-1 [23]	%	5.0	±0.27	0.7
Penetration at 25 °C, EN 1426 [24]	0.1·mm	60	±0.9	3.8
Softening point, EN 1427 [25]	°C	50	±0.27	0.3
Fraass breaking point, EN 12,593 [26],	°C	−4.7	±0.65	1.0
Dynamic viscosity at 60 °C, EN 13702-1 [27]	Pa·s	474.7	±20.5	36.0
Elastic recovery, EN 13,398 [28]	%	11.7	±1.7	3.0

**Table 2:** Analysis of the gradation of reclaimed asphalt pavement.

Sieve size (mm)	0.063	0.125	0.50	1.0	2.0	4.0	8.0	16.0	31.5
Passing %	0.4	0.9	6.4	11.2	18.9	32.8	56.5	89.8	100.0

**Table 3:** Properties of recycled cement concrete (RC).

Aggregate		Result	with Continuous Gradation
Grain size	d/D	–	0/63
Gradation and tolerance	Categories	–	GA85, GTA20
Grain shape–flatness indicator	Category	16.0	FI20
Grain shape–shape indicator	Category	13.0	SI20
Amount of crushed and rounded grains	Category	100%	C90/3
Amount of dust	Category	4.2	f5
Crushing resistance	Category	31.0	LA40
Bulk density of grains ρa	Declared value	2.280	2.280 Mg/m^3^
Absorption	Category	4.9	WA_24_4.9
Frost resistance	Category	3.2	F4

**Table 4:** The results of the determination of the geometrical, physical properties of recycled aggregate with a continuous gradation of 0/31.5 mm.

No.	Properties	Test According to	Result	Category According to PN-EN 13,242
1	Gradation	EN 933-1 [30]	-	GA85
2	Flatness index%	EN 933-3 [32]	17	FI_20_
3	Shape index %	EN 933-4 [33]	22	SI_40_
4	Fines content %	EN 933-1 [30]	5.8	f_7_
5	Filler quality MBF g/kg	EN 933-9 [34]	3.8	MB_F_10
6	Crushing resistance—Los Angeles coefficient, (10/14 mm) % *	EN 1097-2 [35]	36	LA_40_
8	Grain density, Mg/m^3^ *–ρa	EN 1097-6 [36]	2.65	declared value
9	Absorption % *	EN 1097-6 [36]	1.2	WA_24_2
10	Frost resistance % *	EN 1367-1 [37]	0.3	F1

*—The tests were carried out on a separated aggregate fraction.

**Table 5:** Properties of 50/70 paving grade bitumen.

Property	Unit	Result
Penetration at 25 °C	0.1 mm	59
Softening point	°C	49.5
Fraass breaking point	°C	−13
Kinetic viscosity at 135 °C	mm^2^/s	488
Dynamic viscosity at 60 °C	Pa·s	290

**Table 6:** Properties of CEM I 42.5R Portland cement.

Property	Testing Method	Unit	Result
Beginning of setting	EN 196-3 [38]	min	182
Compression strength	EN 196-1 [39]		
after 2 days		MPa	31.9
after 28 days		MPa	57.8
Soundness	EN 196-3 [38]	mm	0.6
Specific surface area	EN 196-6 [40]	cm^2^/g	4282

**Table 7:** Composition of recycled mix with recycled aggregate (RA).

Mix	FB	CEM I 42.5 R	RA	VA 0/31.5
	[%]	[%]	[%]	[%]
FB-RCM (80 RA + 20 VA)	2.5	2.0	75.9	17.5
FB-RCM (20 RA + 80 VA)			17.5	75.9

**Table 8:** Composition of recycled mix with reclaimed asphalt pavement (RC).

Mix	FB	CEM I 42.5 R	RAP	VA 0/31.5
	[%]	[%]	[%]	[%]
FB-RCM (80 RAP + 20 VA)	2.5	2.0	78.0	17.5
FB-RCM (60 RAP + 40 VA)			58.5	37.0
FB-RCM (40 RAP + 60 VA)			39.0	56.5
FB-RCM (20 RAP + 80 VA)			17.5	78.0

**Table 9:** Composition of recycled mix with recycled cement concrete (RC).

Mix	FB	CEM I 42. 5R	RC	VA 0/31.5
	[%]	[%]	[%]	[%]
FB-RCM (80 RC + 20 VA)	2.5	2.0	78.0	17.5
FB-RCM (60 RC + 40 VA)			58.5	37.0
FB-RCM (40 RC + 60 VA)			39.0	56.5
FB-RCM (20 RC + 80 VA)			17.5	78.0

**Table 10:** Optimum amount of water in the mineral mix with reclaimed asphalt pavement (RAP).

Mix	Testing Method	Unit	OMC
FB-RCM 20% RAP + 80% VA	EN 13286-2 [47]	%	6.6
FB-RCM 40% RAP + 60% VA			6.1
FB-RCM 60% RAP + 40% VA			5.8
FB-RCM 80% RAP + 20% VA			5.6
FB-RCM 20% RC + 80% VA			5.8
FB-RCM 40% RC + 60% VA			6.1
FB-RCM 60% RC + 40% VA			6.6
FB-RCM 80% RC + 20% VA			7.3
FB-RCM 20% RA + 80% VA			5.5
FB-RCM 80% RA + 20% VA			5.7

**Table 11:** Testing methods used for the assessment of the FB-RCM mix.

No.	Property	Test Standard
1.	air void content (Vm)	EN 12697-8 [15]
2.	axial compression strength (Rc)	EN 13286-41 [16]
3.	indirect tensile strength (ITS)	EN 12697-23 [17]
4.	water resistance (TSR)	Wirtgen [18]
5.	water and frost resistance	AASHTO T 283 [19]
6.	stiffness modulus S_m_	EN 12697-26 [20] (in the IT-CY sample loading arrangement)
7.	dynamic modulus ǀE*ǀ phase shift angle (φ)	EN 12697-26 [20] (in the DTC-CY sample loading arrangement)

**Table 12:** Parameters of adaptation of module E* master curves.

Mix FB-RCM	Model Adaptation Parameters							Module [MPa]		Assessment of Adaptation	
	a	b	α	β	γ	δ	λ	E ∞	E_0_	RMSE [%]	R^2^
20% RAP	1.92	−0.02	1.97	0.22	−0.39	1.97	6.89	93	8713	0.84	0.98
40% RAP	1.62	−0.04	2.07	−0.86	−0.41	2.08	3.15	119	14,377	2.15	0.96
60% RAP	2.57	−0.01	2.15	1.03	−0.22	2.15	0.10	141	19,881	2.63	0.99
80% RAP	2.49	−0.01	2.02	2.01	−0.35	2.02	1.05	103	10,753	1.30	0.99
20% RC	2.57	−0.009	2.03	0.44	−0.19	2.03	0.1	108	11,687	1.0	0.99
40% RC	2.01	−0.017	2.70	0.23	−0.075	2.70	0.1	502	252,230	4.84	0.97
60% RC	2.84	−0.007	2.05	0.86	−0.16	2.05	0.1	113	12,845	1.08	0.98
80% RC	2.03	−0.02	2.19	−0.51	−0.13	2.19	0.1	154	23,726	3.46	0.98
20% RA	2.12	−0.017	2.45	0.25	−0.058	2.45	0.1	258	81,269	2.71	0.97
80% RA	2.17	−0.015	2.56	0.29	−0.06	2.56	0.1	363	131,792	2.58	0.98

## Data Availability

Data available on request from the corresponding author.
